# Crystal structure of 2,5-di­methyl­anilinium hydrogen maleate

**DOI:** 10.1107/S160053681402282X

**Published:** 2014-10-24

**Authors:** Maha Mathlouthi, Daron E. Janzen, Mohamed Rzaigui, Wajda Smirani Sta

**Affiliations:** a Laboratoire de Chimie des Matériaux, Faculté des Sciences de Bizerte, 7021 Zarzouna Bizerte, Tunisia; b Department of Chemistry and Biochemistry, St Catherine University, 2004 Randolph Avenue, #4282, St Paul, MN 55105, USA

**Keywords:** crystal structure, 2,5-di­methyl­anilinium cation, maleate anion, hydrogen bonding

## Abstract

The crystal structure of the title salt, C_8_H_12_N^+^·C_4_H_3_O_4_^−^, consists of a 2,5-di­methyl­anilinium cation and an hydrogen maleate anion. In the anion, a strong intra­molecular O—H⋯O hydrogen bond is observed, leading to an *S*(7) graph-set motif. In the crystal, the cations and anions pack in alternating layers parallel to (001). The ammonium group undergoes inter­molecular N—H⋯O hydrogen-bonding inter­actions with the O atoms of three different hydrogen maleate anions. This results in the formation of ribbons extending parallel to [010] with hydrogen-bonding motifs of the types *R* ^4^_4_(12) and *R* ^4^_4_(18).

## Related literature   

For active pharmaceutical ingredients (API), see: Kelley *et al.* (2013 ▶). An example of the modification of API properties through the change of one of the mol­ecular components is the substitution of the saccharinate anion in the anti-HIV active lamivudine saccharinate by maleate (Martins *et al.*, 2009 ▶). For 2,5-di­methyl­anilinium cations in combination with other anions, see: Smirani & Rzaigui (2009**a* ▶,b* ▶).
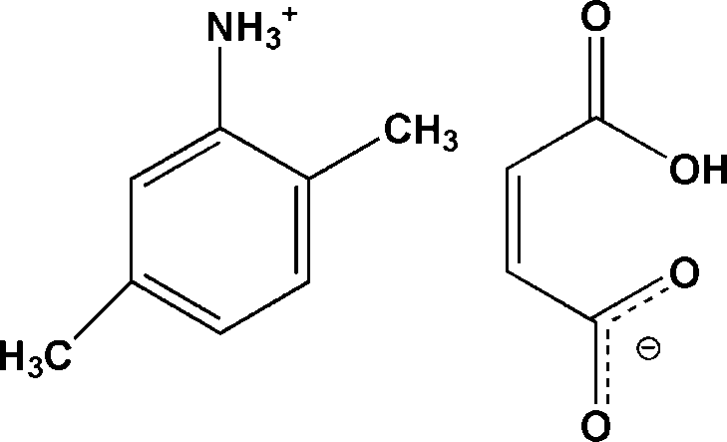


## Experimental   

### Crystal data  

C_8_H_12_N^+^·C_4_H_3_O_4_^−^
*M* *_r_* = 237.25
Triclinic, 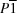

*a* = 6.7983 (17) Å
*b* = 8.515 (2) Å
*c* = 11.012 (3) Å
α = 108.784 (8)°
β = 98.026 (7)°
γ = 98.742 (7)°
*V* = 584.3 (3) Å^3^
*Z* = 2
Mo *K*α radiation
μ = 0.10 mm^−1^
*T* = 173 K
0.45 × 0.26 × 0.19 mm

### Data collection  

Rigaku XtaLAB mini diffractometer
Absorption correction: multi-scan (*REQAB*; Rigaku, 1998 ▶) *T*_min_ = 0.833, *T*_max_ = 0.981
6136 measured reflections
2667 independent reflections
2222 reflections with *F* ^2^ > 2.0σ(*F* ^2^)
*R*_int_ = 0.021

### Refinement  

*R*[*F* ^2^ > 2σ(*F* ^2^)] = 0.042
*wR*(*F* ^2^) = 0.108
*S* = 1.06
2667 reflections
172 parameters
H atoms treated by a mixture of independent and constrained refinement
Δρ_max_ = 0.25 e Å^−3^
Δρ_min_ = −0.28 e Å^−3^

### 

Data collection: *CrystalClear* (Rigaku, 2011 ▶); cell refinement: *CrystalClear*; data reduction: *CrystalClear*; program(s) used to solve structure: *SHELXS97* (Sheldrick, 2008 ▶); program(s) used to refine structure: *SHELXL97* (Sheldrick, 2008 ▶); molecular graphics: *CrystalStructure* (Rigaku, 2011 ▶); software used to prepare material for publication: *CrystalStructure*.

## Supplementary Material

Crystal structure: contains datablock(s) General, I. DOI: 10.1107/S160053681402282X/wm5077sup1.cif

Structure factors: contains datablock(s) I. DOI: 10.1107/S160053681402282X/wm5077Isup2.hkl

**Fig. 1.**
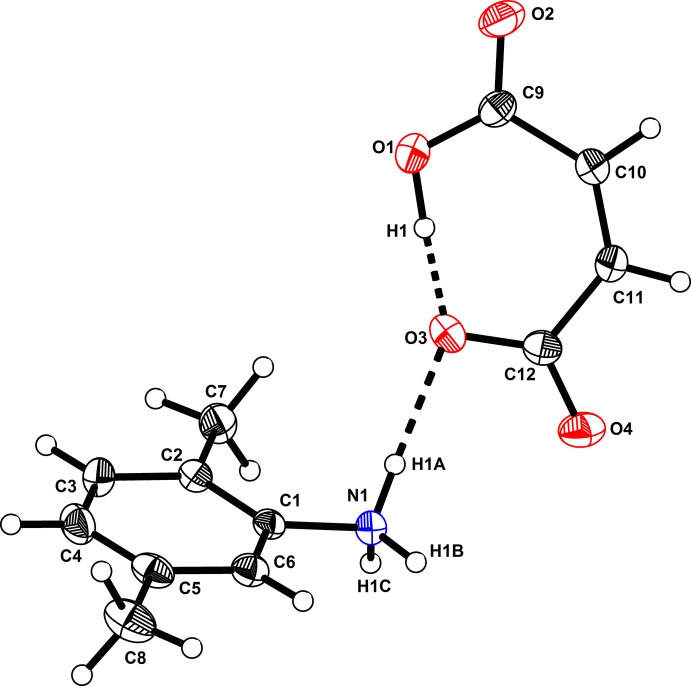
View of the molecular components of 2,5-dimethylanilinium hydrogen maleate with the atom numbering scheme. Displacement ellipsoids for non-H atoms are drawn at the 50% probability level. Hydrogen bonds are shown as dashed lines.

. DOI: 10.1107/S160053681402282X/wm5077fig1.tif
View of the mol­ecular components of 2,5-di­methyl­anilinium hydrogenmaleate with the atom numbering scheme. Displacement ellipsoids for non-H atoms are drawn at the 50% probability level. Hydrogen bonds are shown as dashed lines.

**Fig. 2.**
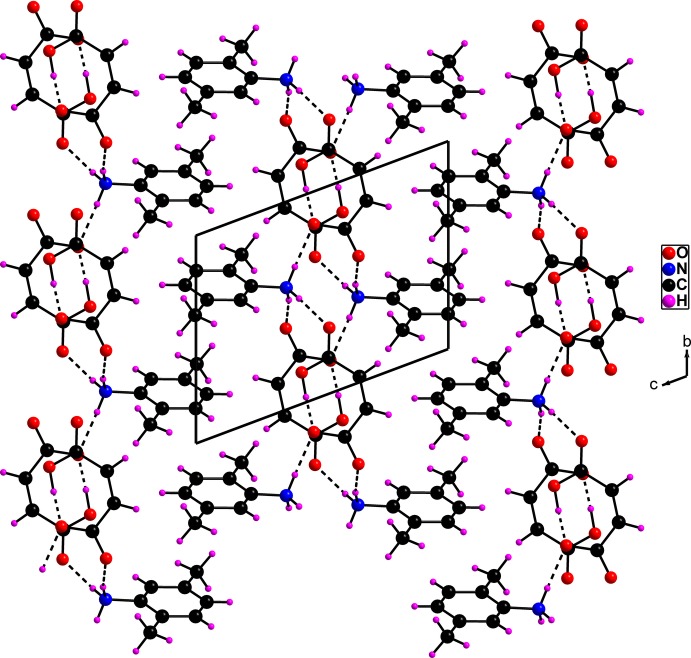
View of the molecular arrangement of the title compound along [100]. Hydrogen bonds are shown as dashed lines.

. DOI: 10.1107/S160053681402282X/wm5077fig2.tif
View of the mol­ecular arrangement of the title compound along [100]. Hydrogen bonds are shown as dashed lines.

**Fig. 3.**
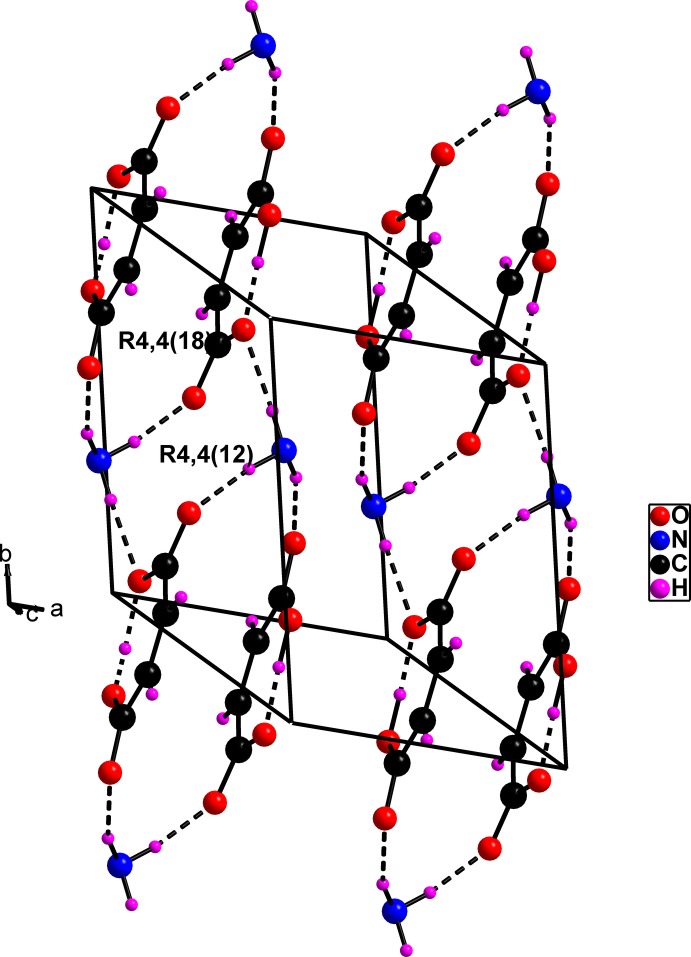
Graph-set description of ring-type hydrogen bonding. Hydrogen bonds are shown as dashed lines.

. DOI: 10.1107/S160053681402282X/wm5077fig3.tif
Graph-set description of ring-type hydrogen bonding. Hydrogen bonds are shown as dashed lines.

CCDC reference: 1029719

Additional supporting information:  crystallographic information; 3D view; checkCIF report

## Figures and Tables

**Table 1:** Hydrogen-bond geometry (, )

*D*H*A*	*D*H	H*A*	*D* *A*	*D*H*A*
O1H1O3	1.02(3)	1.45(3)	2.4651(16)	175(2)
N1H1*A*O3	0.92(3)	1.94(3)	2.859(2)	177.2(15)
N1H1*C*O4^i^	0.92(2)	1.86(2)	2.7602(18)	168(2)
N1H1*B*O2^ii^	0.94(2)	1.88(2)	2.7920(17)	161.4(16)

Symmetry codes: (i) 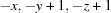; (ii) 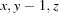.

## supplementary crystallographic information

### S1. Comment

Use of salts and co-crystals of active pharmaceutical ingredients (APIs) as a method for tuning their delivery and activity is an area of growing interest. (Kelley *et al.* (2013)). Modifying API properties such as solubility by finding new salts that employ similar hydrogen-bonding have been successful. A recent example includes increasing the solubility of the anti-HIV drug lamivudine saccharinate by substituting with the anion maleate. (Martins *et al.* (2009)). In an effort to further study the hydrogen-bonding patterns of the maleate ion with other ammonium salts, we report here the synthesis and crystal structure of 2,5-dimethylanilinium maleate.

The structure consists of a protonated 2,5-dimethylanilinium cation with the hydrogen maleate anion (Fig. 1). H1 of the maleate anion undergoes intramolecular O—H···O hydrogen-bonding with O3 as the acceptor. This is common in many structures of maleic acid as the *cis* disposition of the alkene places hydrogen bonding donors and acceptors in close proximity. As such, the maleate anion is very flat (mean deviation from a least-squares plane composed of atoms O1–O4, C9–C12, H1 of 0.04 Å). Parallel maleate anions pack in layers in between layers of parallel 2,5-dimethylanilinium cation layers. The cation layers are parallel with the *ab* plane at *c* = 0. The anion layers are parallel with the *ab* plane at *c* = 1/2 (Fig. 2). The hydrogen atoms of the protonated amine (H1A, H1B, H1C) undergo intermolecular N—H···O hydrogen-bonding interactions with oxygen atoms of three different maleate anions. The two hydrogen-bonding motifs dominating the structure are *R*^4^_4_(12) and *R*^4^_4_(18). These ring motifs form ribbons of hydrogen bonding that are parallel with the *b* axis (Fig. 3).

### S2. Experimental

A mixture of maleic acid (1*M*) and 2,5-xylidine dissolved in ethanol (molar ratio 1:1:1) was stirred for 2 h and then kept at room temperature. Colourless crystals of the title compound were obtained one week later.

### S3. Refinement

H atoms were treated in calculated positions and refined as riding with distances of C—H = 0.95 and 0.98 Å for the phenyl and methyl groups, respectively, and with *U*_iso_(H) = 1.2*U*_eq_(C). Hydrogen atoms bonded to N or O atoms were located in a difference Fourier map, and their positions and *U*_iso_(H) values were refined freely.

### Crystal data

**Table:**

C_8_H_12_N^+^·C_4_H_3_O_4_^−^	*Z* = 2
*M**_r_* = 237.25	*F*(000) = 252.00
Triclinic, *P*1	*D*_x_ = 1.348 Mg m^−^^3^
Hall symbol: -P 1	Mo *K*α radiation, λ = 0.71075 Å
*a* = 6.7983 (17) Å	Cell parameters from 5478 reflections
*b* = 8.515 (2) Å	θ = 3.1–27.7°
*c* = 11.012 (3) Å	µ = 0.10 mm^−^^1^
α = 108.784 (8)°	*T* = 173 K
β = 98.026 (7)°	Prism, colorless
γ = 98.742 (7)°	0.45 × 0.26 × 0.19 mm
*V* = 584.3 (3) Å^3^	

### Data collection

**Table:**

Rigaku XtaLAB mini diffractometer	2222 reflections with *F*^2^ > 2.0σ(*F*^2^)
Detector resolution: 6.849 pixels mm^-1^	*R*_int_ = 0.021
ω scans	θ_max_ = 27.5°
Absorption correction: multi-scan (*REQAB*; Rigaku, 1998)	*h* = −8→8
*T*_min_ = 0.833, *T*_max_ = 0.981	*k* = −11→11
6136 measured reflections	*l* = −14→14
2667 independent reflections	

### Refinement

**Table:**

Refinement on *F*^2^	Secondary atom site location: difference Fourier map
*R*[*F*^2^ > 2σ(*F*^2^)] = 0.042	Hydrogen site location: inferred from neighbouring sites
*wR*(*F*^2^) = 0.108	H atoms treated by a mixture of independent and constrained refinement
*S* = 1.06	*w* = 1/[σ^2^(*F*_o_^2^) + (0.0485*P*)^2^ + 0.1991*P*] where *P* = (*F*_o_^2^ + 2*F*_c_^2^)/3
2667 reflections	(Δ/σ)_max_ < 0.001
172 parameters	Δρ_max_ = 0.25 e Å^−^^3^
0 restraints	Δρ_min_ = −0.28 e Å^−^^3^
Primary atom site location: structure-invariant direct methods	

### Special details

**Table:**

Geometry. ENTER SPECIAL DETAILS OF THE MOLECULAR GEOMETRY
Refinement. Refinement was performed using all reflections. The weighted *R*-factor (*wR*) and goodness of fit (*S*) are based on *F*^2^. *R*-factor (gt) are based on *F*. The threshold expression of *F*^2^ > 2.0 σ(*F*^2^) is used only for calculating *R*-factor (gt).

### Fractional atomic coordinates and isotropic or equivalent isotropic displacement parameters (Å^2^)

**Table:**

	*x*	*y*	*z*	*U*_iso_*/*U*_eq_	
O1	0.28044 (17)	1.15154 (14)	0.58091 (10)	0.0323 (3)	
O2	0.37703 (18)	1.31701 (13)	0.47170 (11)	0.0355 (3)	
O3	0.19359 (18)	0.84302 (14)	0.53298 (10)	0.0329 (3)	
O4	0.11116 (16)	0.60871 (13)	0.35616 (11)	0.0330 (3)	
N1	0.25093 (19)	0.58663 (15)	0.64194 (11)	0.0215 (3)	
C1	0.34834 (19)	0.65824 (16)	0.78115 (12)	0.0196 (3)	
C2	0.2577 (2)	0.77054 (16)	0.86650 (13)	0.0215 (3)	
C3	0.3508 (3)	0.83054 (18)	0.99864 (13)	0.0267 (3)	
C4	0.5245 (3)	0.78132 (19)	1.04275 (14)	0.0295 (4)	
C5	0.6148 (2)	0.67116 (18)	0.95572 (15)	0.0264 (3)	
C6	0.5240 (2)	0.60942 (17)	0.82285 (14)	0.0231 (3)	
C7	0.0705 (3)	0.82651 (19)	0.81904 (14)	0.0279 (3)	
C8	0.8045 (3)	0.6188 (3)	1.00350 (18)	0.0371 (4)	
C9	0.3196 (2)	1.17476 (17)	0.47453 (14)	0.0240 (3)	
C10	0.2959 (3)	1.02714 (18)	0.35196 (14)	0.0261 (3)	
C11	0.2359 (3)	0.86040 (17)	0.32482 (13)	0.0253 (3)	
C12	0.1743 (2)	0.76436 (17)	0.41028 (14)	0.0236 (3)	
H1	0.240 (4)	1.025 (4)	0.564 (3)	0.070 (8)*	
H1A	0.237 (3)	0.671 (3)	0.6082 (18)	0.033 (5)*	
H3	0.2938	0.9073	1.0602	0.0320*	
H4	0.5826	0.8235	1.1337	0.0354*	
H1C	0.124 (3)	0.523 (3)	0.6319 (19)	0.041 (5)*	
H6	0.5823	0.5341	0.7611	0.0277*	
H7A	−0.0376	0.7266	0.7696	0.0334*	
H7B	0.1026	0.8909	0.7623	0.0334*	
H7C	0.0251	0.8984	0.8943	0.0334*	
H8A	0.7767	0.5596	1.0642	0.0445*	
H8B	0.9130	0.7198	1.0486	0.0445*	
H8C	0.8472	0.5430	0.9287	0.0445*	
H1B	0.321 (3)	0.511 (3)	0.5915 (19)	0.036 (5)*	
H10	0.3294	1.0566	0.2801	0.0313*	
H11	0.2317	0.7919	0.2369	0.0304*	

### Atomic displacement parameters (Å^2^)

**Table:**

	*U*^11^	*U*^22^	*U*^33^	*U*^12^	*U*^13^	*U*^23^
O1	0.0469 (7)	0.0269 (6)	0.0224 (6)	0.0110 (5)	0.0078 (5)	0.0058 (5)
O2	0.0427 (7)	0.0205 (6)	0.0417 (7)	0.0047 (5)	0.0167 (6)	0.0062 (5)
O3	0.0478 (7)	0.0312 (6)	0.0248 (6)	0.0102 (5)	0.0101 (5)	0.0148 (5)
O4	0.0339 (6)	0.0226 (6)	0.0416 (7)	0.0012 (5)	0.0088 (5)	0.0118 (5)
N1	0.0251 (6)	0.0209 (6)	0.0179 (6)	0.0041 (5)	0.0043 (5)	0.0064 (5)
C1	0.0217 (7)	0.0179 (6)	0.0191 (7)	0.0000 (5)	0.0043 (5)	0.0082 (5)
C2	0.0233 (7)	0.0208 (7)	0.0214 (7)	0.0037 (5)	0.0059 (6)	0.0088 (6)
C3	0.0329 (8)	0.0264 (7)	0.0195 (7)	0.0053 (6)	0.0071 (6)	0.0060 (6)
C4	0.0320 (8)	0.0317 (8)	0.0209 (7)	−0.0020 (6)	−0.0014 (6)	0.0108 (6)
C5	0.0214 (7)	0.0279 (7)	0.0322 (8)	−0.0001 (6)	0.0016 (6)	0.0174 (7)
C6	0.0227 (7)	0.0223 (7)	0.0268 (7)	0.0044 (5)	0.0071 (6)	0.0111 (6)
C7	0.0294 (8)	0.0313 (8)	0.0269 (8)	0.0129 (6)	0.0088 (6)	0.0112 (6)
C8	0.0251 (8)	0.0449 (10)	0.0471 (10)	0.0044 (7)	0.0004 (7)	0.0281 (8)
C9	0.0216 (7)	0.0225 (7)	0.0263 (7)	0.0069 (6)	0.0047 (6)	0.0056 (6)
C10	0.0326 (8)	0.0250 (7)	0.0217 (7)	0.0046 (6)	0.0071 (6)	0.0095 (6)
C11	0.0308 (8)	0.0229 (7)	0.0195 (7)	0.0034 (6)	0.0043 (6)	0.0051 (6)
C12	0.0210 (7)	0.0239 (7)	0.0276 (8)	0.0060 (5)	0.0042 (6)	0.0110 (6)

### Geometric parameters (Å, º)

**Table:**

O1—C9	1.305 (2)	C4—C5	1.391 (3)
O1—H1	1.02 (3)	C4—H4	0.9500
O2—C9	1.227 (2)	C5—C6	1.397 (2)
O3—C12	1.2777 (18)	C5—C8	1.509 (3)
O4—C12	1.2422 (17)	C6—H6	0.9500
N1—C1	1.4671 (17)	C7—H7A	0.9800
N1—H1A	0.922 (19)	C7—H7B	0.9800
N1—H1C	0.92 (2)	C7—H7C	0.9800
N1—H1B	0.941 (19)	C8—H8A	0.9800
C1—C2	1.3926 (19)	C8—H8B	0.9800
C1—C6	1.389 (2)	C8—H8C	0.9800
C2—C3	1.3946 (19)	C9—C10	1.4876 (19)
C2—C7	1.510 (3)	C10—C11	1.337 (2)
C3—C4	1.388 (3)	C11—C12	1.494 (3)
C3—H3	0.9500		
			
C9—O1—H1	109.8 (14)	C5—C6—H6	120.0
C12—O3—H1	111.1 (9)	C2—C7—H7A	109.5
C1—N1—H1A	111.0 (11)	C2—C7—H7B	109.5
C1—N1—H1C	109.7 (12)	H7A—C7—H7B	109.5
H1A—N1—H1C	108.3 (16)	C2—C7—H7C	109.5
C1—N1—H1B	112.6 (11)	H7A—C7—H7C	109.5
H1A—N1—H1B	109.7 (15)	H7B—C7—H7C	109.5
H1C—N1—H1B	105.3 (16)	C5—C8—H8A	109.5
C6—C1—C2	122.76 (12)	C5—C8—H8B	109.5
C6—C1—N1	119.04 (12)	H8A—C8—H8B	109.5
C2—C1—N1	118.19 (12)	C5—C8—H8C	109.5
C1—C2—C3	116.33 (12)	H8A—C8—H8C	109.5
C1—C2—C7	122.09 (12)	H8B—C8—H8C	109.5
C3—C2—C7	121.57 (12)	O2—C9—O1	121.78 (13)
C4—C3—C2	121.87 (13)	O2—C9—C10	117.86 (13)
C4—C3—H3	119.1	O1—C9—C10	120.36 (12)
C2—C3—H3	119.1	C11—C10—C9	131.55 (13)
C3—C4—C5	120.95 (13)	C11—C10—H10	114.2
C3—C4—H4	119.5	C9—C10—H10	114.2
C5—C4—H4	119.5	C10—C11—C12	130.50 (13)
C4—C5—C6	118.13 (13)	C10—C11—H11	114.8
C4—C5—C8	120.96 (14)	C12—C11—H11	114.8
C6—C5—C8	120.91 (14)	O4—C12—O3	123.77 (13)
C1—C6—C5	119.94 (13)	O4—C12—C11	116.61 (13)
C1—C6—H6	120.0	O3—C12—C11	119.59 (12)
			
C6—C1—C2—C3	−1.19 (19)	C2—C1—C6—C5	1.2 (2)
N1—C1—C2—C3	177.61 (12)	N1—C1—C6—C5	−177.58 (12)
C6—C1—C2—C7	178.07 (12)	C4—C5—C6—C1	−0.11 (19)
N1—C1—C2—C7	−3.14 (18)	C8—C5—C6—C1	179.49 (12)
C1—C2—C3—C4	0.1 (2)	O2—C9—C10—C11	−179.46 (15)
C7—C2—C3—C4	−179.14 (13)	O1—C9—C10—C11	0.7 (2)
C2—C3—C4—C5	0.9 (2)	C9—C10—C11—C12	−1.0 (3)
C3—C4—C5—C6	−0.9 (2)	C10—C11—C12—O4	175.04 (15)
C3—C4—C5—C8	179.48 (13)	C10—C11—C12—O3	−6.8 (2)

### Hydrogen-bond geometry (Å, º)

**Table:**

*D*—H···*A*	*D*—H	H···*A*	*D*···*A*	*D*—H···*A*
O1—H1···O3	1.02 (3)	1.45 (3)	2.4651 (16)	175 (2)
N1—H1*A*···O3	0.92 (3)	1.94 (3)	2.859 (2)	177.2 (15)
N1—H1*C*···O4^i^	0.92 (2)	1.86 (2)	2.7602 (18)	168 (2)
N1—H1*B*···O2^ii^	0.94 (2)	1.88 (2)	2.7920 (17)	161.4 (16)

Symmetry codes: (i) −*x*, −*y*+1, −*z*+1; (ii) *x*, *y*−1, *z*.
