# Supplementary figures and images for: Crystal structure of 2,5-di­methyl­anilinium hydrogen maleate
Source: Acta Crystallogr Sect E Struct Rep Online. 2014 Oct 24;70(Pt 11):o1183–4. doi: 10.1107/S160053681402282X (PMC4257352; doi:10.1107/S160053681402282X)

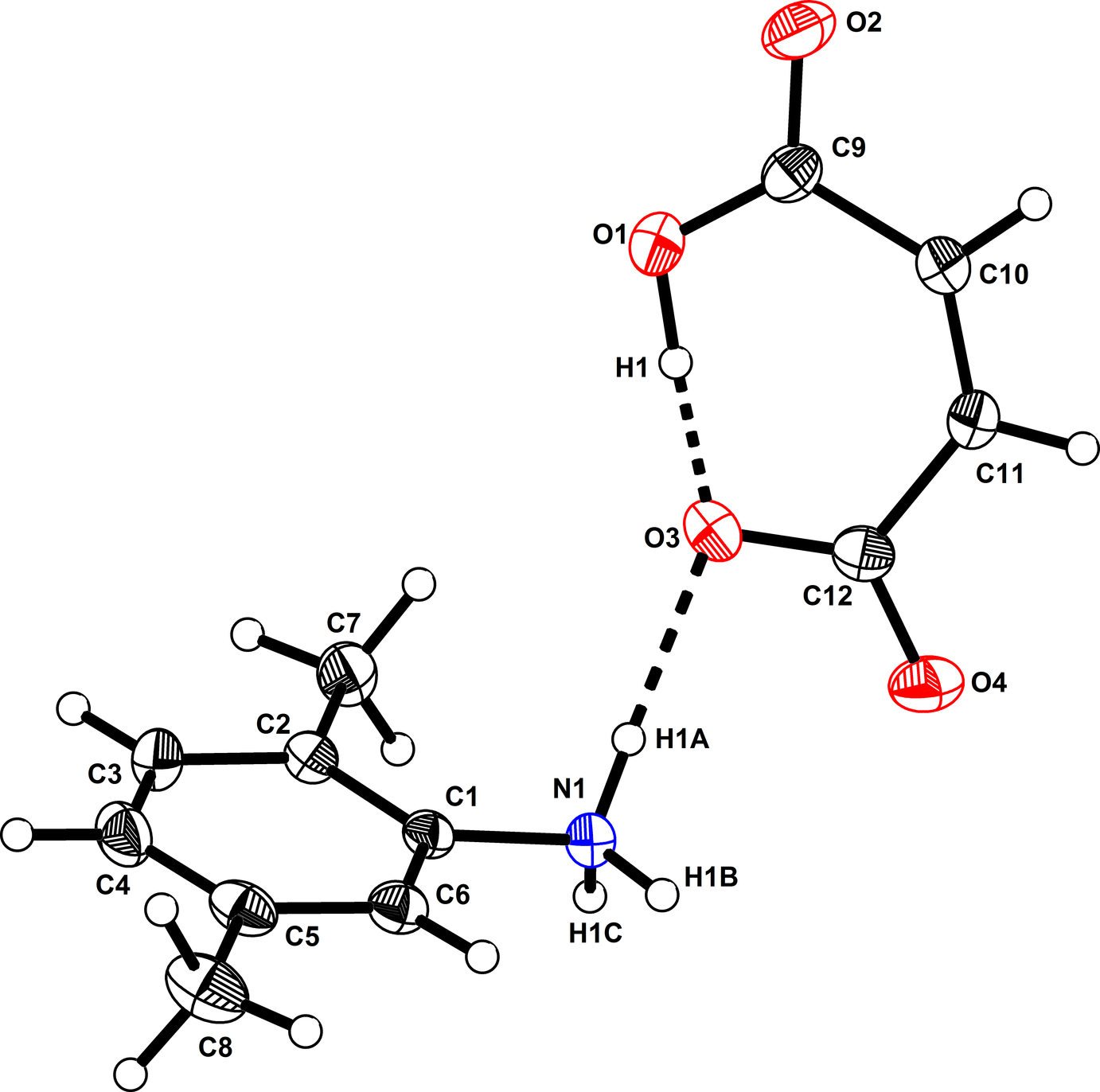

Supplement: Supplementary file 3 [file e-70-o1183-fig1.tif]

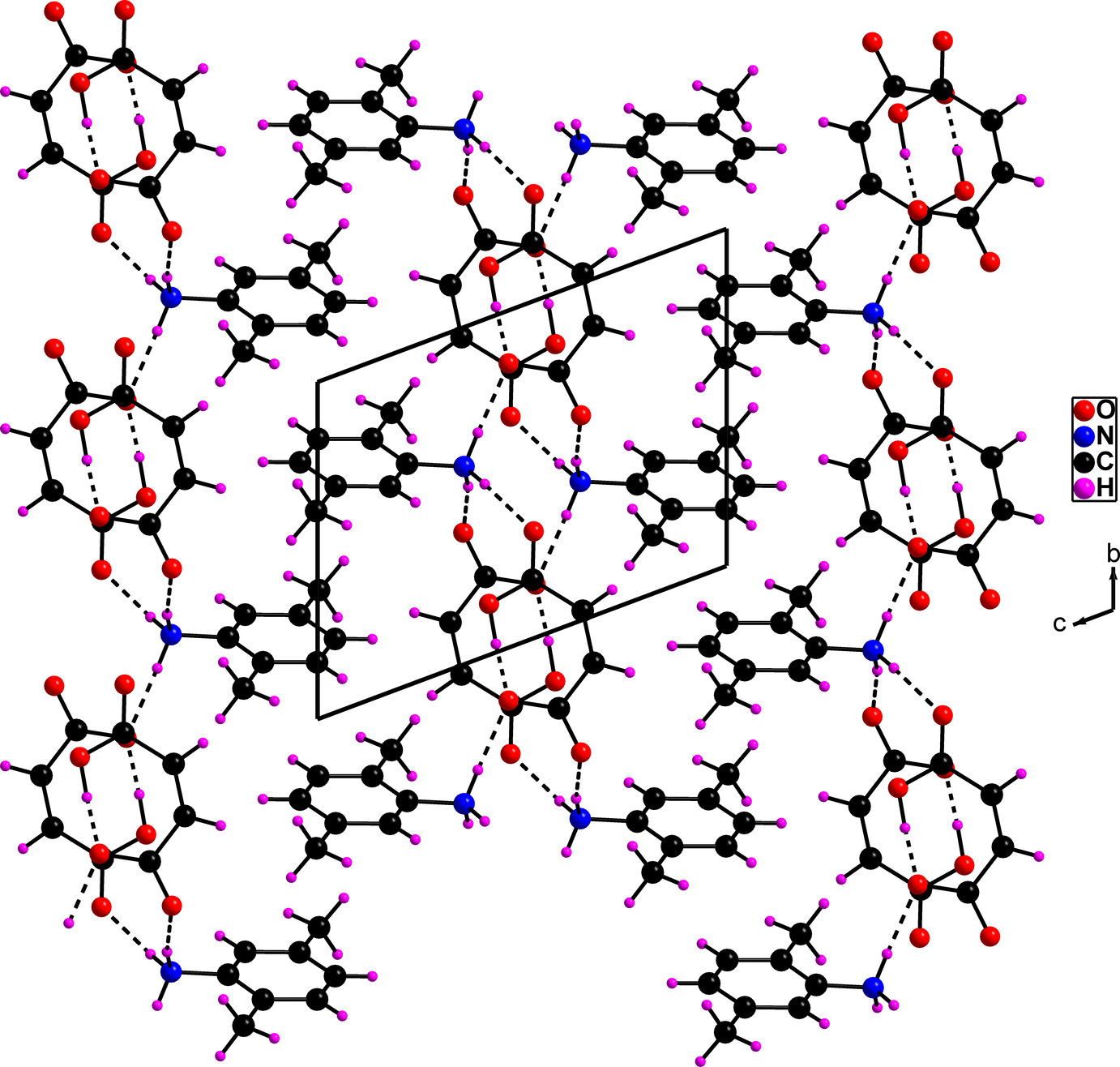

Supplement: Supplementary file 4 [file e-70-o1183-fig2.tif]

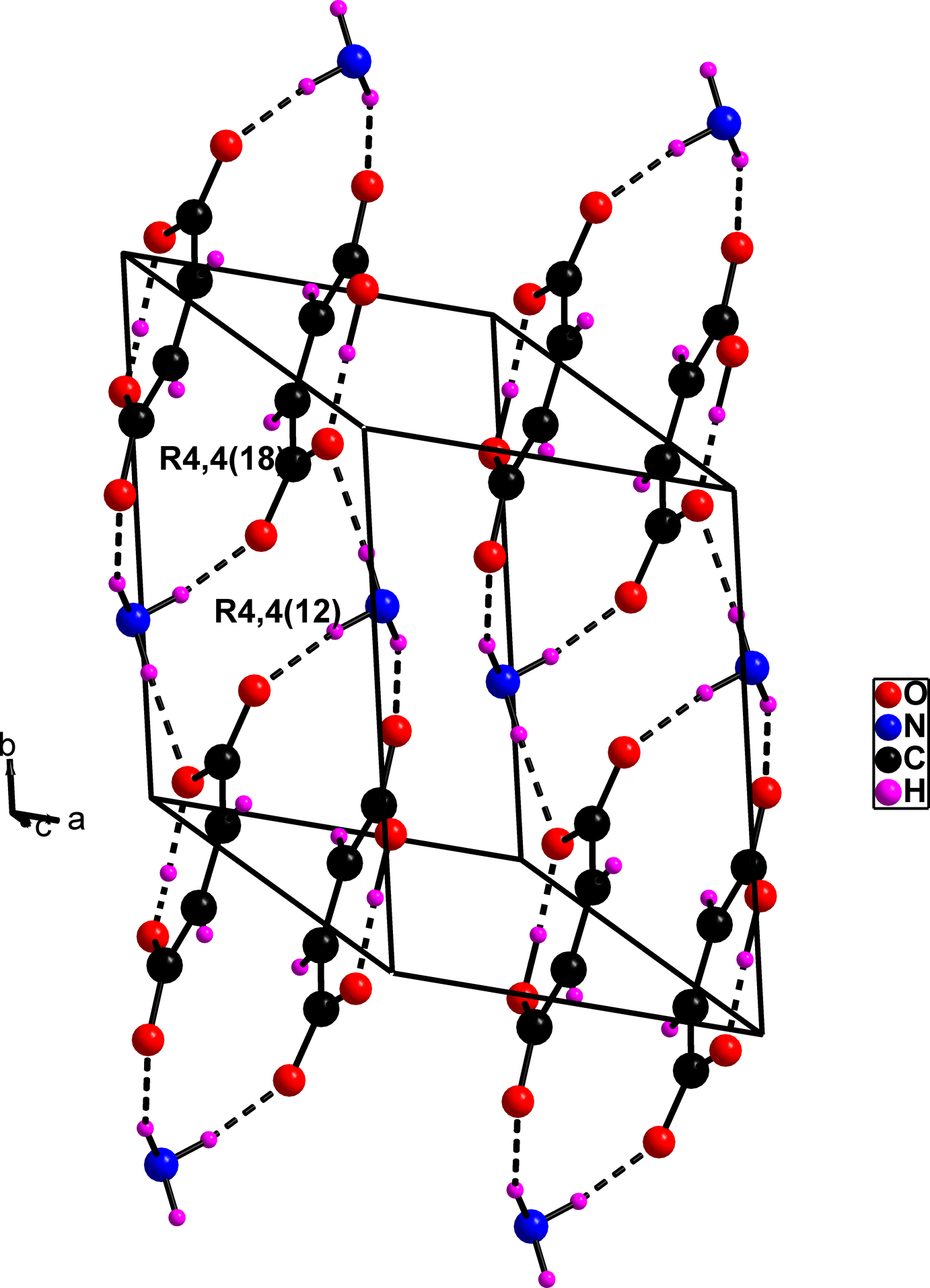

Supplement: Supplementary file 5 [file e-70-o1183-fig3.tif]
